# Total Mercury in Yellow Knights (*Tricholoma equestre*) Mushrooms and Beneath Soils

**DOI:** 10.1007/s00128-012-0757-x

**Published:** 2012-08-07

**Authors:** Dawid Maćkiewicz, Jerzy Falandysz

**Affiliations:** Institute of Environmental Sciences and Public Health, University of Gdańsk, 18 Sobieskiego Str., 80-952 Gdańsk, Poland

**Keywords:** Foods, Forest, Heavy metals, Organic food, Pollution, *Suillus grevillei*, Trace metals, Wild food

## Abstract

Mercury has been determined in caps, stipes and whole fruiting bodies of Yellow Knights mushrooms and the beneath top layer of soils from ten geographically distant locations in Poland. The Yellow Knights can be considered as an effective accumulator of total Hg. The mean values of bioconcentration factor (BCF) of Hg in caps of Yellow Knights for nine of the locations ranged from 22 ± 9 to 75 ± 13 (total range 9.0–90) and for stipes from 13 ± 7 to 52 ± 9 (total 4.4–93). The top layer (0–10 cm) of soils in these nine sites contained Hg with mean (±SD) concentration ranging from 0.019 ± 0.003 to 0.046 ± 0.007 ng/g dry weight. Mercury was less accumulated (BCF 4.9 ± 2.7 for a whole fruiting bodies) by Yellow Knights that emerged at the most contaminated site, where soil contained 0.059 ± 0.028 ng Hg/g dw. The potential of Yellow Knights communities to bioconcentrate Hg (determined as BCF) in fruiting bodies varied between the localities more than tenfold and decreased highly with increase of Hg content of the top soil.

Mercury emissions and diffusion in the environment and contamination of food resources remain an actual problem of debate and concern worldwide. Wild grown mushrooms are highly vulnerable to Hg contamination (Falandysz et al. 2001a; Melgar et al. 2009). One of the reasons for elevated Hg content of forest mushrooms can be the degree of forest soil’s pollution with this element (Suchara and Sucharová 2002). Wild grown mushrooms can be very effective accumulators of Hg and some of them highly bioconcentrated methyl mercury (Chudzyński et al. 2009, 2011; Drewnowska et al. 2012a, b; Falandysz 2012; Falandysz et al. 2012a, b; Grgić et al. 1992; Stijve and Roschnik 1974).

Yellow Knight *Tricholoma equestre,* mushroom, synonymous to “Man on Horseback” is specie popular during the fall and early winter and is widely distributed in Europe and North America (Læssøe et al. 1996). This mushroom is known also as *Tricholoma flavovirens* that is mycorrhizal with the conifer trees – especially of the *Pinus* family, and is found on sandy soils. In Poland the Yellow Knight is traditionally regarded as tasty mushroom, which is suitable for drying, pickling, stewing, souring and freezing. In a recipe on mushroom soups, “the Yellow Knights’ soup”, it is noted that the fresh specimens do not need to be blanched before soup cooking – that is the specimens, when “cleaned up, cut and well rinsed in running tap water, are poured directly into boiling bullion and boiled for 40 min”. A common practice is blanching of mushrooms (a short-time boiling) by boiling them for 2, 3, 5 or 10 min in boiling and slightly salty water – depending on the gourmet recipe. The Yellow Knight is officially registered as edible mushroom in Poland. There are some recent controversies on its edibility especially when consumed for several days in large amount. Because of this, in Germany, this mushroom is actually classified in the group of dangerous species.

This study documents the pollution of forest soil by Hg as well as the evaluating the Hg content, bioconcentration and distribution in *T*. *equestre* mushrooms collected from ten spatially distant sites in the Central Europe country of Poland. None of these sites surveyed was recognized as being under the influence of local Hg pollution.

## Materials and Methods

The Yellow Knights, *T*. *equestre* (L.) P. Kumm., 1871, mushrooms and soil top layer (0–10 cm; ca. 100 g) beneath to the fruiting bodies were collected from ten spatially distant areas across Poland in 1999–2007 (Table 1).

**Table 1:** Total mercury concentrations in caps and stipes of *T. equestre* and beneath soil (μg/g dw), BCF and cap to stipe Hg content ration quotients (Q_C/S_)

Land of Poland, site, year of collection; number of specimens	Hg			Q_C/S_	BCF	
	Caps	Stipes	Soil		Cap	Stipe
Pomerania, Hel Peninsula, Hel, 2004; n = 15	0.96 ± 0.32	0.62 ± 0.23	0.046 ± 0.007	1.7 ± 0.7	22 ± 9	14 ± 5
	(0.87)	(0.60)	(0.045)	(1.4)	(21)	(13)
	0.45–1.5	0.36–1.2	0.038–0.059	0.59–3.9	9.0–37	6.1–26
Pomerania, Kolbudy Forest Inspectorate, Otomin, 2006; n = 13 (29)	0.77 ± 0.21	0.56 ± 0.26	0.019 ± 0.003	1.6 ± 0.5	41 ± 12	29 ± 13
	(0.71)	(0.49)	(0.020)	(1.7)	(40)	(27)
	0.49–1.3	0.25–0.98	0.012–0.022	0.94–2.3	23–60	11–51
Pomerania, Rzecznica, 2003; n = 10	0.97 ± 0.10	0.69 ± 0.12	0.013 ± 0.002	1.4 ± 0.2	75 ± 13	52 ± 9
	(0.96)	(0.69)	(0.013)	(1.4)	(75)	(51)
	0.83–1.1	0.53–0.85	0.010–0.016	1.3–1.9	59–90	39–68
Kociewie Land, Tucholskie forest, Łuby, 2001; n = 14	0.85 ± 0.06	0.65 ± 0.10	0.019 ± 0.003	1.3 ± 0.2	47 ± 12	36 ± 9
	(0.82)	(0.64)	(0.018)	(1.3)	(45)	(35)
	0.62–1.3	0.52–0.86	0.013–0.025	0.83–1.8	33–72	24–58
Kujawy Land, Ciechocinek, 2004; n = 15	1.3 ± 0.7	1.1 ± 0.8	0.037 ± 0.002	1.2 ± 0.3	35 ± 21	31 ± 23
	(0.99)	(0.80)	(0.037)	(1.1)	(27)	(23)
	0.57–2.8	0.54–3.3	0.035–0.039	0.86–2.1	15–81	15–93
Kujawsko-Pomorskie Voivodeship, Brzoza, 1999; n = 14	0.71 ± 0.11	0.68 ± 0.14	0.028 ± 0.006	1.1 ± 0.1	26 ± 6	25 ± 7
	(0.72)	(0.71)	(0.026)	(1.1)	(25)	(25)
	0.50–0.85	0.43–0.91	0.022–0.039	0.83–1.2	18–38	15–35
Podlasie Land, Augustów, 1998/1999; n = 11	0.25 ± 0.07	0.17 ± 0.04	0.036 ± 0.009	1.5 ± 0.2	24 ± 8	20 ± 7
	(0.24)	(0.16)	(0.037)	(1.5)	(20)	(17)
	0.17–0.36	0.11–0.23	0.021–0.047	1.2–1.8	14–40	14–31
Podlasie Land, Augustów, 2006; n = 15	0.81 ± 0.10	0.63 ± 0.06	0.035 ± 0.001	1.3 ± 1.3	25 ± 8	19 ± 6
	(0.80)	(0.63)	(0.037)	(1.3)	(20)	(17)
	0.68–0.94	0.53–0.74	0.021–0.047	1.0–1.5	15–40	14–31
Mazowsze Land, Commune of Kościelna Wieczfnia, 2006; n = 13	0.23 ± 0.06^a^		0.059 ± 0.028	NA	4.9 ± 2.7	
	(0.21)		(0.060)		(4.0)	
	0.16–0.32		0.031–0.13		(1.2–9.6)	
Lubelskie Land, Chodelska Dale, Poniatowa, 1999–2001; n = 13	0.84 ± 0.42	0.49 ± 0.30	0.039 ± 0.007	1.9 ± 1.8	22 ± 12	13 ± 7
	(0.56)	(0.45)	(0.036)	(1.8)	(18)	(13)
	0.46–1.8	0.15–1.1	0.030–0.050	1.0–3.6	9.3–56	4.4–25

*NA* not applicable

^a^Whole fruiting bodies

The fruiting bodies after careful cleanup from the plant material and particles of soil with a plastic knife were rinsed with distilled water and oven dried at 65°C to constant weight. Dried caps and stipes of fruiting bodies were milled in porcelain mortar and further kept in brand new sealed polyethylene bags in clean condition until chemical analysis. Soil substrate samples, after removal of the visible organisms, small stones, sticks and leaves were air dried under clean condition at room temperature for approximately 6 weeks and then sieved through a pore size of 2 mm.

Total Hg content of materials was determined by cold-vapour atomic absorption spectroscopy (CV-AAS). The samples matrix was pyrolised and the released mercury was first amalgamated and then desorbed from gold wool (Mercury analyzer type MA-2000, Nippon Instruments Corporation, Takatsuki, Japan). Analytical control and assurance quality (AC/AQ) were achieved by the analysis of several reference standard materials (Jarzyńska and Falandysz 2011). One of these materials was the CS-M-1 certified reference material (dried fruiting bodies of mushroom Cow Bolete (*Suillus bovinus*) produced by the Institute of Nuclear Chemistry and Technology in Warsaw, Poland. This material is routinely used in the analysis performance check-up and its’ declared total Hg content is 0.174 ± 0.018 μg/g dw. In this study, Hg content of the CS-M-1 certified reference material was 0.176 ± 0.004 μg/g dw (n = 3). With every set of 10 mushroom or soil samples examined one blank sample was run and no interference was noted. The analytical equipment under the condition used enabled the determination of Hg at linear range spanning from 0.002 to 1,000 ng in a sample of up to 0.5 g mass. Discrepancies between the certified values and concentrations quantified were well below 10 %. Duplicates and blanks followed with every set of ten samples examined. For blank samples, no major interferences were found for the elements quantified. Limit of detection for Hg was 0.005 μg Hg/g dw. Coefficients of variation for these measurements on routine runs were well below 10 %.

All data produced were statistically treated using the computer software Statistica version 8.0.

## Results and Discussion

Data on Hg content of top layer of forest soils and fruiting bodies of *T*. *equestre* as well as on the Hg distribution between caps and stipes and the Hg bioconcentration factor (BCF) values by species are presented in Table 1.

The unit less BCF value is used as a measure of accumulation potential of elements by mushroom. The BCF is a quotient of chemical element content in fruiting body against its content in soil substratum. The BCF values of Hg in caps and stipes of *T*. *equestre* were rather high for most sites and did indicate a strong potential of this mushroom to sequestrate Hg. BCF up to 90 was noted in a single cap, while median values of BCF varied for most of the sites between 18 and 75; the mean values ranged from 22 ± 9 to 75 ± 13 (Table 1). In the Commune Kościelna Wieczfnia of the Mława County, the mean value of BCF was 4.9 ± 2.7 (median was 4), and this is distinctly low compared to other areas in this study. Mercury content of top layer of forest soil in the Commune Kościelna Wieczfnia was higher compared to other sites examined, i.e. 0.059 ± 0.028 μg/g dw, while median for nine other sites varied between 0.013 and 0.045 μg/g dw, and the means ranged from 0.019 ± 0.003 to 0.046 ± 0.007 ng/g (Table 1). Nevertheless, these data on Hg in forest soils do not show any on pollution problem and could be considered as “baseline” levels.

Mercury content was higher in caps compared to stipes of *T*. *equestre*. The median values of the quotient of Hg content in cap to Hg content in stipe (Q_C/S_) varied from 1.1 to 1.8 (Table 1). In this study, for seven of the sites examined Hg content of caps ranged from 0.71 ± 0.11 to 1.3 ± 0.7 μg/g dw (median values were from 0.72 to 0.99 μg/g dw) and for two other sites in one case in caps was 0.25 ± 0.07 μg/g dw (median 0.24 μg/g dw) and in another (in a whole fruiting body) was 0.23 ± 0.06 μg/g dw (median 0.21 μg/g dw) (Table 1). In some earlier studies of *T*. *equestre*, the specimens examined contained Hg in caps of 0.94 ± 0.74 μg/g dw (n = 3; 1994) and 1.7 ± 0.9 μg/g dw (n = 14; 1996–1997) (Falandysz et al. 2001b, 2003). Based on data on total Hg in *T*. *equestre* in this and earlier studies it can be concluded that a site to site variation of Hg content over years can be up to ten-fold, while the upper mean and median content in caps did not exceed 2.0 μg/g dw.

Mercury accumulated in fruiting bodies of mushrooms might pose health risk for consumers. In countries where *T*. *equestre* grows, it could be eaten by some individuals in relatively large amount within a week. Intoxication was noted after eating 1,200 g (fresh weight) fruiting bodies of *T*. *equestre* in 5 days by a boy of age 5 years (Chodorowski et al. 2003). Locally, in autumn in pine forests *T*. *equestre* can be very abundant. This mushroom, after blanching (boiled, pickled) or without blanching (salted) are usually eaten at a rate of around 300–400 g daily – during 3–4 days in a week.

The Hg reference dose (RfD) of 0.0003 mg/kg body weight/day and provisional tolerable weekly intake value of 0.004 mg/kg bw for person of 70 kg bw can be applied to assess health risk to consumers due to Hg taken from foods (JECFA 2010; US EPA 1987).

A meal made of 300 or 400 g fresh caps or whole fruiting bodies of *T*. *equestre* containing 0.24–0.25 μg Hg/g dw on the average (two sites, Table 1) could provide from 0.0072–0.0075 to 0.096–0.010 mg Hg (assuming 90 % of moisture in fresh mushrooms). For eight other sites, where the mean value of Hg in caps were higher, i.e. between 0.71 and 1.3 μg Hg/g dw, a mushroom dish of similar volume could provide from 0.021–0.039 to 0.028–0.052 mg Hg, on the average. These values, calculated for a person of 60-kg body weight gave a range of 0.00035 and 0.00087 mg/kg bw, which are doses exceeding the RfD of Hg and show elevated risks from the consumption of this mushroom. On the other site Hg intake from such meals eaten seven times in a week will provide from 0.149 to 0.199 and from 0.27 to 0.36 mg Hg, and these are intakes equivalent to 2.1–2.8 and 3.9–5.2 μg Hg/kg bw respectively. For some of the sites examined (Table 1) Hg intake solely from caps of *T*. *equestre* is close to or above the PTWI of Hg.

As reported in Table 1, the Yellow Knights, even if they emerged at sites unpolluted with Hg, are mushroom species that are usually contaminated with elevated concentrations of this element, which is a feature common to certain other edible wild mushrooms (Falandysz et al. 2002, 2004, 2007a, b).

Data on methylmercury (CH_3_Hg^+^) content and proportion to total Hg in *T*. *equestre* are not available. Selenium in foods is considered as element that to some degree is also able to reduce or eliminate toxicity from Hg and CH_3_Hg^+^. Mercury can form species hardly soluble in water such as HgSe and HgS but nothing is known on such Hg compounds in mushrooms. Knowledge of Se and Hg interactions in flesh of mushrooms is also non-existing. Selenium content of *T*. *equestre* is known only from a single study and the reported Se concentration of 2.8 ± 0.3 (2.5–3.2; n = 4) μg/g dw seems overestimated (cited after Falandysz 2008).
